# Dissection of genetic architecture of rice plant height and heading date by multiple-strategy-based association studies

**DOI:** 10.1038/srep29718

**Published:** 2016-07-13

**Authors:** Liyuan Zhou, Shouye Liu, Weixun Wu, Daibo Chen, Xiaodeng Zhan, Aike Zhu, Yingxin Zhang, Shihua Cheng, Liyong Cao, Xiangyang Lou, Haiming Xu

**Affiliations:** 1 Institute of Crop Science and Institute of Bioinformatics, College of Agriculture and Biotechnology, Zhejiang University, Hangzhou 310058, China; 2 State Key Laboratory of Rice Biology and Zhejiang Key Laboratory of Super Rice Research, China National Rice Research Institute, Hangzhou 311401, China; 3 Department of Biostatistics and Bioinformatics, Tulane University, New Orleans, Louisiana, 1440 Canal St., Suite 2001, New Orleans, LA 70112-2632, USA

## Abstract

Xieyou9308 is a certified super hybrid rice cultivar with a high grain yield. To investigate its underlying genetic basis of high yield potential, a recombinant inbred line (RIL) population derived from the cross between the maintainer line XieqingzaoB (XQZB) and the restorer line Zhonghui9308 (ZH9308) was constructed for identification of quantitative trait SNPs (QTSs) associated with two important agronomic traits, plant height (PH) and heading date (HD). By re-sequencing of 138 recombinant inbred lines (RILs), a total of ~0.7 million SNPs were identified for the association studies on the PH and HD. Three association mapping strategies (including hypothesis-free genome-wide association and its two complementary hypothesis-engaged ones, QTL-based association and gene-based association) were adopted for data analysis. Using a saturated mixed linear model including epistasis and environmental interaction, we identified a total of 31 QTSs associated with either the PH or the HD. The total estimated heritability across three analyses ranged from 37.22% to 45.63% and from 37.53% to 55.96% for the PH and HD, respectively. In this study we examined the feasibility of association studies in an experimental population (RIL) and identified several common loci through multiple strategies which could be preferred candidates for further research.

As an important staple crop that provides food source for over half of the world population, rice (*Oryza sativa* L.) plays a pivotal role in food security in most rice growing countries especially in Asia^1^. To satisfy the food demand, super rice breeding program was launched in China in 1996^2^. Since then dozens of super rice varieties including Xieyou9308 have been successfully bred and a significant advance has been achieved after release of these super rice varieties to rice farming^2^. However, for further improvement, it is a necessary process to dissect the genetic basis of valuable agronomic traits. Xieyou9308 is a super hybrid rice variety with a grain yield as high as 12.23 ton/ha^2^ and thus attracts more attention toward uncovering of its genetic mechanisms involved in high yield potential. Plant height (PH) and heading date (HD) are two determinant traits owing to their crucial roles in plant architecture and environment adaptability that are closely associated with the yield potential of rice.

In general, the PH and HD are regarded as complex traits which are quantitatively inherited. Based on the conventional molecular markers and experimental population, many studies on QTL linkage mapping have been conducted to explore the casual loci for the phenotypic variation of the PH and HD in various rice populations as recorded in the Gramene QTL Database (http://archive.gramene.org/qtl/)^3^, including several QTL linkage studies based on derived populations from Xieyou9308 for dissecting the genetic basis of important agronomic traits^4^,^5^,^6^. Although numerous QTLs have been identified, only a minority of them has been successfully cloned because the detected QTLs still cover a large candidate region where there are considerable number of unrelated genes^7^ due to the limited number of polymorphism markers used and the insufficient meiotic recombination events in conventional linkage mapping. As a result, a lot of work on narrowing down of the QTLs is still needed until it can be applied in consequent breeding program effectively, such as marker-assisted selection (MAS). Association study is another promising analytic approach that makes use of historic recombination events for mapping casual genes, but are limited in the early time because of the unavailability of sufficient number of polymorphisms. More recently, benefiting from the advances on the genotyping technology, it has become possible and commonplace to obtain a large panel of polymorphisms such as the single nucleotide polymorphisms (SNPs), which results in extensive applications of association methods in genetic dissection of complex traits on a genome-wide scale for various organisms^8^,^9^,^10^,^11^ including rice^12^,^13^,^14^. Such new technologies provide the promise to accelerate detection and cloning of QTLs and thus to conduct precise molecular breeding design for improvement of many crop quantitative traits^15^,^16^. Using association strategy, Huang *et al*.^12^ conducted a genome-wide mapping in a natural population of *Oryza sativa indica* subspecies and identified a total of 37 loci associated with 14 agronomic traits, including 7 loci for HD. Moreover, a GWAS based on 413 diverse accessions of *O. sativa* from 82 countries, identified 234 loci associated with 34 agronomic traits using 44,100 identified SNP variants^13^. In a further investigation by Huang *et al*.^14^, they reported other 32 new loci associated with flowering time and with ten grain-related traits based on a natural population of 950 worldwide rice varieties including the *Oryza sativa indica* and *Oryza sativa japonica* subspecies.

In the present study, to explore the specific genetic basis of the super hybrid rice Xieyou9308 and to further dissect QTL regions for mining functional genes, a recombinant inbred line (RIL) population derived from the Xieyou9308 was sequenced for the identification of quantitative trait SNPs (QTSs) associated with the PH and HD by using a newly developed association mapping software, *QTXNetwork*^17^. It was developed based on mixed linear model approaches, and includes GMDR-GPU^18^ as a module for efficient preliminary filtering when necessary. Additionally, to make full use of information from previous studies (including previously reported QTLs and annotated genes of rice) and thus improve the reliability of the detected QTSs, three mapping strategies, i.e., the “agnostic” genome-wide association mapping (GWA) that treats all genetic variants equally across the whole genome ignoring prior information, and two complementary hypothesis-driven strategies: QTL-based association mapping (QBA) and gene-based association mapping (GBA), were performed separately in this study. The results demonstrated that association mapping in an experimental population could enhance or complement previous QTL mappings through multiple comparative analyses and thus provide more precise genetic variant information for subsequent gene cloning and genetic improvement of the traits.

## Results

### Structure of Linkage Disequilibrium

**Figure 1:**
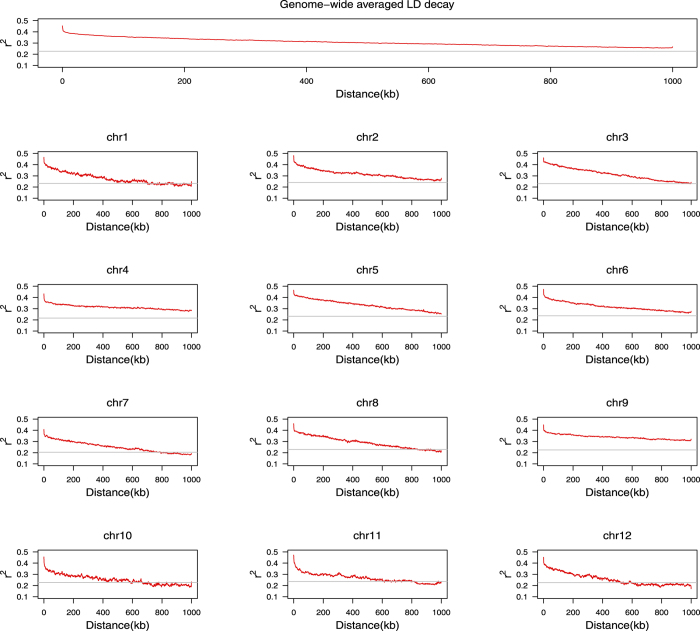
The patterns of LD decay with distance. Decay of LD (*r*^2^) with distance (kb) between SNPs was summarized both across whole genome and each chromosome; the average LD over chromosomes was given in distance 1 kb; the threshold (grey horizontal line) was set at the half of its maximum 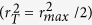.

Linkage disequilibrium (LD) is the basis of association mapping which is also called as linkage disequilibrium mapping (LDM). The extent of LD highly varies across species, populations, even chromosome regions, and thus it is more reliable to estimate the empirical LD according to the investigated population. Here the LD decay rate is measured at the chromosomal distance when the average squared pairwise correlation coefficient (*r*^2^) of SNPs drops to the half of its maximum value 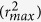. Using PLINK^19^ and the ~0.7 million SNPs, we estimated the pairwise LD for all SNPs in the same chromosome within 1 Mb and then averaged the *r*^2^ every 1 kb distance. The LD patterns were analyzed both on whole-genome wide scale and each separate chromosome and the results are presented in Fig. 1. As shown in the top panel of Fig. 1, the whole genome-wide LD decay rate was estimated at approximately 1,000 kb, which is larger than the previous estimate^12^,^20^ from several rice natural populations (~100 kb to over 200 kb). We further examined the LD patterns of each chromosome and found it varied across 12 chromosomes (Fig. 1) with an estimated value ranging from ~400 kb (chromosome 10) to over 1000 kb (chromosome 4 and chromosome 9), which might lead to differential resolutions at different chromosomes. It is noteworthy that the estimate of LD may also depend on the marker numbers used in calculation as it turned out to be <50 kb when we calculated the pairwise *r*^2^ for every specific SNP only with its 10 adjacent SNPs (the default setting for − −ld− window of PLINK).

### Estimated Heritability and Genetic Effects

Three mapping strategies (GWA, QBA and GBA) were adopted to identify candidate variants responsible for the phenotypic variation of the PH and HD; as a result, a total of 31 QTSs were detected significantly associated with the target traits. As shown in Table 1, the total phenotypic variance explained by the corresponding SNPs varies from 37.22% to 45.63% and from 37.53% to 55.95% for the PH and the HD, respectively. There was no significant QTS-environment interaction detected, indicating that the two traits are genetically highly stable across the two investigated locations. In addition, no epistasis was detected by three strategies for the PH and HD (Table 1).

**Figure 2:**
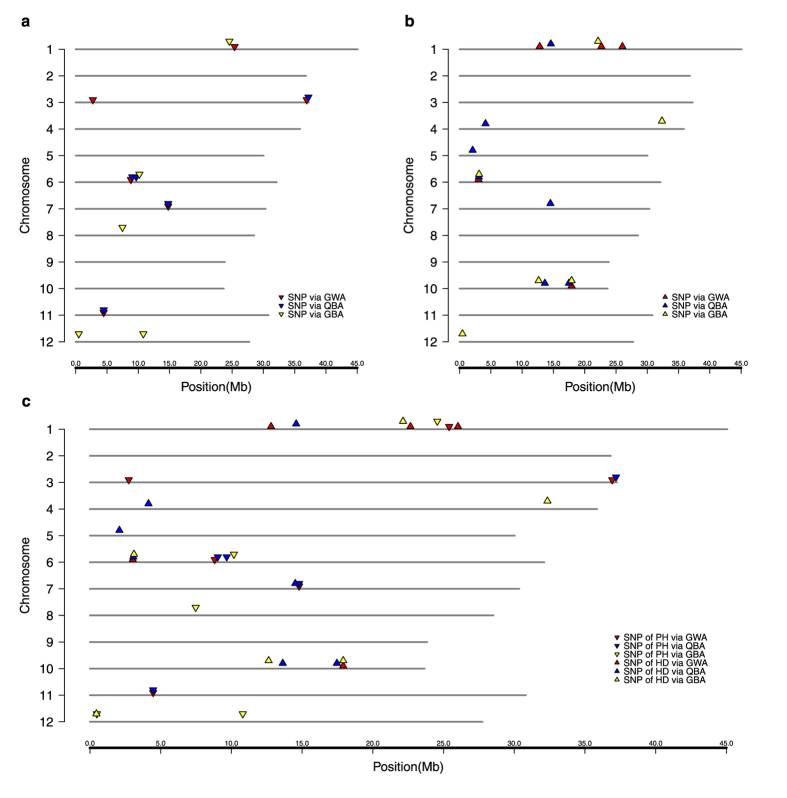
The genomic distribution of detected QTSs for the PH and the HD. (**a**) Genomic positions of detected significant QTSs associated with the plant height through three strategies: Red, genome-wide association strategy (GWA); Blue, QTL-based association strategy (QBA); Yellow, gene-based association strategy (GBA). (**b**) Genomic positions of detected significant QTSs associated with the heading date through three strategies. (**c**) Genomic positions of detected significant QTSs associated with the plant height (Inverted triangle) and the heading date (triangle) through three strategies.

As shown in Table 1, 14 QTSs were identified for the PH by three strategies, of which 6 were detected by the GWA, 5 by the QBA and 5 by the GBA, respectively. In the GWA mapping, the estimated total heritability of 6 SNPs was 45.63% and four of these SNPs exhibited large and positive additive effects with individual heritability ranging from 4.62% to 11.99%. Of note, the positive or negative effects of loci depend on the coding of genetic variants. In this study we coded the additive coefficient of paternal allele homozygotes (QQ, ZH9308) as “1” and the maternal allele homozygotes (qq, XQZB) as “−1”. The reported effect is for the genotype of QQ and it should be the opposite value for genotype of qq. Here the positive additive effects indicate that paternal allele homozygotes (QQ, ZH9308) at these loci (rs25397447, rs2745731, rs36929057, rs8819284) could increase the plant height while it will be decreased by their maternal allele homozygotes genotype (qq, XQZB) at these loci; in contrast, the plant height will be increased by its maternal allele homozygote (qq, XQZB) at the SNP rs14788399 and the rs4468159 because they exhibited negative additive effects (Table 1). The following mapping results could be interpreted in the similar way. In the QBA mapping, we detected 5 significant SNPs distributing on 4 chromosomes with the total estimated heritability of 37.22%, less than that in the GWA mapping. Likewise, two SNPs (rs14788399 and rs4468159) which were also declared significant in the GWA still exhibited negative additive effects while the other three SNPs (rs37194796, rs9040677, rs9669459) expressed positive additive effects with individual heritability ranging from 6.1% to 9.89%. In the GBA mapping, we obtained the total heritability of 40.19% contributed by 5 SNPs with individual heritability varying from 5.72% to 10.63%; out of these detected SNPs, only one SNP (rs10809004) showed negative effects while the others exhibited positive additive. In comparison of results from three strategies, we observed there are four physically close SNPs (rs8819284, rs9040677, rs9669459 and rs10179007) on chromosome 6 identified by three mapping strategies (Fig. 2a); three of them (rs8819284, rs9040677 and rs10179007) have relatively stronger pairwise LD (*r*^2^) between each other (ranging from 0.52 to 0.56, see Supplementary Fig. S1), which indicate these three SNPs may represent one same causal locus controlling the PH; the remaining one (rs9669459) has very weak pairwise LD (*r*^2^) with the other three SNPs (ranging from 0.006 to 0.02), indicating it should be another genetic variant although it is quite physically near the other three SNPs and also exhibits similar magnitude additive effect on the PH as rs9040677 in the QBA (Table 1). In addition, there were three loci, scattering in the end of chromosome 3, the middle of chromosome 7 and the forehead of chromosome 11, detected by two strategies (GWA and QBA, Fig. 2a). For the loci located on chromosome 7 and chromosome 11, they were captured by the rs14788399 on chromosome 7 and by the rs4468159 on chromosome 11, respectively, which were detected both in the GWA and the QBA; the remaining locus located on chromosome 3 was captured by different SNPs (rs36929057 in GWA and rs37194796 in QBA), but with pairwise LD (*r*^2^) reaching up to 0.78, indicating these two SNPs might still represent one same causal locus. These common loci detected in multiple strategies could be regarded as high priority candidates for follow-up.

For the HD (Table 1), we totally identified 17 significant SNPs; out of them, only the rs17909546 on chromosome 10 was detected by both the GWA and the GBA. In the GWA mapping, 5 significant SNPs were detected with total heritability of 41.56%: only the rs3033880 on chromosome 6 showed large negative additive effects with individual heritability of 11.53%; two SNPs (rs22665688, rs17909546) showed similar modest-size positive additive effects while the remaining two (rs12807091, rs26023433) showed relatively small positive additive effects. In the QBA mapping, we obtained the largest heritability (55.95%) contributed by 7 SNPs: as we would expect, there was one locus on chromosome 6 captured by SNP rs3070122 showing negative additive effect with heritability of 11.55%, which was also captured by another SNP rs3033880 with similar effect size and heritability (11.53%) in the GWA; additionally, we found that another locus on chromosome 5 captured by SNP rs2083119 also exhibited negative additive effect; the remaining five SNPs showed modest-size positive additive effects with individual heritability ranging from 5.72% to 9.53%. In the GBA mapping, we obtained the total estimated heritability of 37.53% explained by 6 SNPs: we found the locus located on chromosome 6, which was detected previously both in the GWA and QBA, still showed negative effects at SNP rs3112796; the other SNPs exhibited positive additive effects with individual heritability varying from 4.09% to 9.79%. Likewise, we obtained some common or approximately common loci across multiple strategies (Fig. 2b). The SNP rs17909546 on chromosome 10 is an exactly common one detected both in the GWA and GBA and its adjacent SNP (rs17462383) was detected in the QBA with pairwise LD (*r*^2^) of 0.40 (see Supplementary Fig. S1). The aforementioned locus on chromosome 6 with negative effects is an approximately common locus because it was captured by three different SNPs (rs3033880, rs3070122, rs3112796) in three strategies between which there exist strong pairwise LD (*r*^2^) ranging from 0.34 to 0.53. In addition, there are other two loci located on chromosome 1, one of which was captured by rs12807091 in the GWA and by rs14583697 in the QBA (with LD (*r*^2^) of 0.53), and the other was captured by rs22665688 in the GWA and by rs22139712 in the GBA (with LD (*r*^2^) of 0.46). As described above, these common or approximately common loci detected by at least two strategies could be regarded as preferred candidates for further research.

It has been observed that two loci resided in the middle of chromosome 7 and the front-end of chromosome 12 are associated with both traits, respectively (Fig. 1c). Generally, these loci shared by multiple traits are regarded as the genetic basis of pleiotropism, which make it possible for us to improve multiple traits simultaneously via manipulating these pleiotropic loci. However, when we examine the correlations between the significant SNPs detected for the PH and DH, only the correlation of the SNPs on chromosome 12 is quite strong (*r*^2^ ≈ 0.52, see Supplementary Fig. S1) but very weak for the SNPs on chromosome 7 (*r*^2^ ≈ 0.003), indicating that the SNPs on chromosome 7 represent actually two different genetic variants though they are quite close in terms of physical distance. For the locus on chromosome 12, the high LD between the representative SNPs and the strong phenotypic correlation between PH and HD (*r* ≈ 0.59, see Supplementary Table S1) together indicated that this locus might have pleiotropic effects.

### Breeding Value Prediction and Candidate Genes

Theoretically, the genetic information on the detected QTSs could be utilized in improving the performance of corresponding target traits in breeding. Base on the genetic effects of QTSs, we could design any target genotypes and evaluate their breeding potential by analysis of genetic values. Since the GE interactions were not detected on the PH and HD in this study, thus the total genetic values keep unchanged across environments. As shown in Table 2, we calculated the total genetic values of the restorer line ZH9308 and the maternal line XQZB whose genotypes at all detected QTSs were denoted as QQ and qq, respectively. Further, the total genetic values were also estimated for all inbred lines, of which the highest one is regarded as the best line and denoted as BL. In addition, using the prediction methods^21^, we also predicted the superior genotype which could achieve the potential highest total genetic values and thus was referred to as superior line (SL). The corresponding estimated population means were also presented in Table 2. As shown in Table 2, the total genetic values of ZH9308 are much higher than XQZB on both traits across three strategies, which is consistent with the fact that the XQZB has inferior performance on plant height and heading date compared with the ZH9308. Moreover, there is no difference between the BL and the predicted SL on total genetic values for the PH and HD across three strategies, meanwhile, both of which are much higher than the better parent (ZH9308), indicating that the selected best recombinant-inbred progeny could improve plant height and heading date as compared with better parent line (ZH9308) but has no potential for further improvement of these traits based on the detected QTSs as it already achieved the potential highest total genetic values.

According to the latest version of the rice genome annotation (MSU Rice Genome Annotation Project Release 7, http://rice.plantbiology.msu.edu/index.shtml), of 31 QTSs associated with the PH and the HD, 15 QTSs are located within annotated genes as presented in Table 3. Most target genes encode constitutive proteins or enzymes, which exert a fundamental role in life activity, like transcription regulation, lipid metabolism, cellular growth and differentiation. For the PH, the variant on chromosome 1 (rs24562025) locates in the gene CENP-C1 (also called CENP-C), which encodes the protein centromere protein C1 (CENPC1/CENPC). This protein is the component of the CENPA-NAC (nucleosome-associated) complex, a complex that plays a role in assembly of kinetochore proteins, mitotic progression and chromosome segregation^22^,^23^. Another plant height associated SNP rs4468159 locates in the gene encoding the DnaK (Hsp70) family protein which is a kind of chaperones protein. Mutations in DnaK have been previously shown to affect both DNA and RNA synthesis in *E. coli*^24^, indicating the DnaK is involved in chromosomal DNA replication. These biological processes may occur during cell proliferation and cell division which are closely related to plant growth. The helix-loop-helix (HLH) proteins harboring the variant rs22139712 associated with the HD has been reported by previous studies^25^ that it functions in developmental processes. GDSL-like lipase/acylhydrolase (harboring rs12807091), retrotransposon protein of Ty3-gypsy subclass (harboring rs12634148) and zinc finger, C3HC4 type domain containing protein (harboring rs457324) also have been reported by previous studies^26^,^27^ that they are closely associated with the heading date. Other detected genes encode protein or enzymes like double-strand break repair protein MRE11, transposon protein etc. which may also influence target traits via complex metabolic network.

## Discussion

More recently, genome-wide association studies (GWAS) has become a powerful tool for mapping causal gene with fast advances in high-throughput genotyping and sequencing technology. Generally, natural population is the first choice for GWAS because of its convenience to collect, abundant accumulated historical recombinant events which is important to achieve much higher mapping resolution. However, the unknown and complex population structure among the collected samples which would lead to the increased false positives, has always been haunting. For most plant species, many designed experiment mapping populations have been constructed for QTL linkage mapping of complex traits, and some of these resources may be also good materials to conduct association mapping when genome-wide high density markers are available because these controlled populations are well-praised for their explicit population structure so that the haunting issue on population heterogeneity could be avoided. However, a widely supposed weakness of controlled populations would be the limited recombinant events which might result in lower mapping resolution compared with natural population. In the present study, as we showed on LD decay rate, the resolution of the experiment population (RIL) is not greatly inferior to that of the natural population (~1/10 to ~1/5). Compared with DH and F2, RIL is more desirable to reduce LD because RIL accumulates more recombinant events than DH and F2 during producing^28^ and thus could present an acceptable resolution in association mapping.

Most phenotypic traits of economic importance in plants, such as the PH and HD, are complex traits which are final performance of jointly interacting networks of multiple genes and environmental factors^29^. In the high-throughput era, hypothesis-free genome-wide association (GWA) is an intuitive strategy for dissecting genetic architecture of complex traits. However, most current GWA studies (GWASs), which generally exhaustively scan whole genome to detect any significant signal based on simple additive model, are unable to detect interaction of paired genes and gene by environment; furthermore, some genetic effects from ignored epistasis and GE interaction will inflate residual error, as a result, the difficulty to identify minor-effect variant will be exacerbated. In the present study, based on mixed linear model approach, we used a saturated model including both epistasis and environmental interaction to detect responsible genetic variants and further estimate genetic effects of significant QTS. This mixed linear model approach is time-consuming; although the software *QTXNetwork*^17^ has been speeded up using parallel computation based on GPU and CPU, it still could not be directly applied to GWAS with a large number of SNPs; thus, we first used GMDR-GPU^18^ to quickly filter candidate SNPs potentially associated with surveyed trait when the number of SNPs is too large (such as GWA and QBA strategy in this study). On the other hand, hypothesis-engaged candidate association strategy could be an alternative option to alleviate the computational burden issue when necessary; because it can dramatically decrease testing candidates and multiple-testing penalty directly by integrating the information from previous studies. The previous detected QTLs in the same experimental population are valuable and association based on the specific QTL regions could largely tackle the aforementioned problem resulting from dense markers. However, the performance of this method largely depends on the reliability of previous QTL studies. Annotated gene-based association (GBA) is also acceptable since it is genes not a single SNP inside involved in the genetics and development of a trait; in addition, it is much easier to understand the biological function of the detected variants in the region of annotated genes. Similarly, this strategy also has its limitation to capture the causal variants in non-coding region where some important regulatory elements may reside. This study provides a comprehensive insight into the genetic basis of the PH and HD in Xieyou9308, and employment of three association mapping strategies helps us identify more genetic variants and the detected consistent variants could be regarded as the preferred candidates for future study and utilization in breeding program. All four preferred candidate loci of the PH are in the region of the reported QTLs from previous linkage analysis in the same genetic materials^30^ and two of which (with representative SNPs of rs10179007 and rs4468159) are in the annotated genes (Table 3). All four preferred candidate loci (captured by the representative SNPs of rs12807091, rs22139712, rs3112796 and rs17909546) of the HD reside in the annotated genes (Table 3), therein, two (rs12807091 and rs3112796) are located in the QTL region^30^ and one (rs17909546) is quite near a QTL region^30^. rs22139712 on chromosome 1 is the new detected preferred candidate by the GWA and the GBA. rs457324 and rs489068 on chromosome 12 detected only by the GBA for the HD and PH, respectively, are the representative SNPs of the only one possible pleiotropic locus associated with the PH and HD (Fig. 2c).

As shown in Fig. 2a, all the loci detected by the QBA (blue) for the PH are quite reliable since they could be confirmed by the other one or two strategies, which indicates that previous linkage analysis are very informative and the QBA is an efficient fine-mapping strategy to narrow down the QTL using the same mapping population. As for the QBA mapping of the HD (blue loci in Fig. 2b), besides the “reliable” loci that could be confirmed by other strategy, we detected three additional loci separately distributing on chromosome 4, 5 and 7, which may, at least in part, contribute to the discrepancy of heritability between the QBA and the GWA/GBA (Table 1). This indicates that the previous detected QTLs are more informative and some relatively minor-effect variants in these QTL regions are easier to identify by the QBA than by the GWA and the GBA benefiting from partial alleviation on the issue of multiple-testing because of relatively small number of SNPs tested in analysis. On the other hand, this also shows there is a risk to lose some true causal variants in the procedures of the GWA and the GBA because some representative SNPs in these QTLs may not be captured in the preliminary filtering on total SNPs. However, this does not mean that the results from the QBA must be superior to that from the GWA or the GBA as it still depends on the accuracy of previous QTLs. For instance, in the GWA and the GBA, we also identified one novel preferred candidate (LOC_Os01g39330) for the HD that is not in the previously reported QTL region. In conclusion, none of the strategies is perfect to dissect and get a comprehensive insight into the intricate genetic architecture of complex traits, but they are complementary to each other in identifying more genetic variants and confirming consistency of the detected variants which could be regarded as preferred ones in future study and utilization in the molecular improvement of target trait. Our study also demonstrated that association mapping in experiment population could enhance or complement previous QTL studies through multiple strategies and thus promote identification of functional genes in QTLs and their application in breeding program.

Even though we used a saturated model including epistasis and gene by environmental interaction to dissect the phenotypic variation, no interactions were detected. It is consistent with the previous QTL studies^5^ which also did not detect epistatic QTLs involved in additive by additive interaction. Further, when we checked the predicted total genetic values (*μ* + *G*) of superior line which is an optimized pure line with homozygous genotype of QQ or qq at each SNP loci, we found that they were 95.80 cm, 93.67 cm and 93.59 cm for the PH through three strategies, respectively. These predicted values are far lower than the average performance of the Xieyou9308 (~120 cm for the PH) which is the F1 hybrid of bi-parents with genotype consisting of many heterozygous loci. It is suggested that the allelic interactions (dominance) or their related epistasis interactions might play a crucial role in the genetic basis of these two target traits; therefore, for further investigation of this genetic architecture, a recombinant inbred intercross (RIX) population which is generated from random mating of recombinant inbred (RI) strains would be ideal since it contains more heterogeneous loci as well as more kinds of combination of two genes in different positions on genome, which are basic requirements for analyzing dominance, dominance-related epistasis effects and their interaction effects with environment, and then we might observe more epistasis or environmental interactions.

## Methods

### Plant Materials and Field Experiments

The RIL mapping population used in this study consists of 138 F_13_ lines, which are derived from the super hybrid rice cross between the maintainer line XieqingzaoB (XQZB) and the restorer line Zhonghui9308 (ZH9308) through single seed descent method. Now these RILs are maintained in China National Rice Research Institute (CNRRI), which can be accessed with the permission of CNRRI.

The phenotypic experiments were conducted at two experimental farms of CNRRI: One is located at Hangzhou city (120.2 E, 30.3 N), Zhejiang province, China, and the other is located at Lingshui county (110.0 E, 18.5 N), Hainan province, China (see Supplementary Fig. S2). Due to the different climate conditions between two investigated locations, the experiments were conducted in a different time in 2009 for accessing corresponding desired rice-growing conditions. In Hangzhou experimental farm, the germinated seeds were sown in the seeding nursery on May 10th and seedlings were transplanted to field on June 5th, while they were December 3th for seeding and January 6th, 2010 for transplanting in Hainan. For both the field trials, the transplanted seedlings were planted in plots of four-row with 8 plants per row, in randomized complete blocks with two replicates. The distance between plants within row was 20 cm, and the row spacing was 15 cm. Both field trials followed the normal field management, being treated with natural submergence of intermediate water depth throughout the growing season, with normal fertilization for the equivalence of 225 kg N (nitrogen) ha^−1^, 127.5 kg P (phosphorus) ha^−1^ and 330 kg K (potassium) ha^−1^ (N:P:K ≈ 2:1:3), and with normal pest control focusing on Rice Scirpophaga incertulas (walker) and Nilaparvata lugens (stal). The average monthly temperatures were 22.9–29.6 °C in Hangzhou during cultivation (May to September 2009), and 20.7–26.1 °C in Hainan (December 2009 to April 2010), respectively. The photoperiod from seeding to all heading were ranged from 12.6 hL (light)/11.4 hD (dark) to 14.1 hL/9.9 hD in Hangzhou, and it was 11.0 hL/13.0 hD to 12.3 hL/11.7 hD in Hainan, respectively.

Six plants in middle of each plot were sampled for phenotype investigation. The PH was measured by the distance from the soil surface to the top of the panicle of the tallest tiller (in centimeters) and the HD was measured by the duration from the seeding to the showing of the main panicle (in days). The phenotypic data at each location were summarized in Supplementary Table S1, and both the traits approximately followed the normal distribution (see Supplementary Fig. S3).

### SNP Genotyping and Filtering

DNA re-sequencing was conducted by the Beijing Genome Institute (BGI) for two parents with 10X coverage and 138 lines with 2X coverage (see Supplementary Table S2 for details of sequencing and mapping data of each RI line and parents). Using the Nipponbare sequence (Os-Nipponbare-Reference-IRGSP-1.0) as the reference genome, sequence alignment was conducted between the sequence reads of each line and the reference genome using the software of BWA, and SNPs were called for each individual using the software of Samtools with the criteria of base quality over 30, mapping quality over 20 and the maximum sequence depth less than 1000. Then, those SNPs whose missing rates exceed 5% were excluded. Thereafter, the chi-square goodness of fit test was employed to exclude SNPs whose genotype frequency deviated from the theoretical distribution of 1:1 at the significance level of 0.01. Finally, a total of 701,867 SNPs were generated from 138 RILs, of which 41.96% were located in genic regions (see Supplementary Table S3). The minor allele frequency (MAF) in our RIL population is larger than 39%. In addition, associations in this population would not be subject to the population structure since these progenies are from the same ancestry (the cross of XQZB and ZH9308) and the relatedness among these lines are essentially evenly distributed (r ≈ 0.5 for any two individuals).

For the GWA, all the originally genotyped SNPs (701,867) were used for association analysis, but the subsets of SNPs in the annotated genes, and in the QTLs regions previously identified for the target traits in this population, were used for the GBA, and the QBA, respectively. For GBA, the gene annotation information was downloaded from the FTP site of Rice Genome Annotation Project (MSU Rice Genome Annotation Project Release 7, http://rice.plantbiology.msu.edu/index.shtml)^31^. The raw data contains 55,986 loci encoding 66,338 gene models (including UTR regions, extrons, and introns); after removing the unmapped data, we got 55,801 loci encoding 66,153 gene models for the subsequent SNP filtration in GBA. Finally, there were 294,498 SNPs filtered for GBA. For QBA, there were 11 and 17 target QTL regions for PH and HD used for SNP filtration (see Supplementary Table S4), and 52,426 and 59,387 SNPs were screened, respectively.

### Genetic Models and Statistical Analysis

A saturated genetic model was adopted for modeling the genetic architecture of complex traits from multi-environment trials, which includes additive effect (a), additive by additive epistasis effect (aa) as the fixed effects and the corresponding environment interaction effects (ae,aae) as the random effects. The genetic model for phenotypic value of the *k*-th genotypes in the *h*-th environment (*y*_*hk*_) can be expressed by the following mixed linear model,


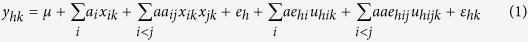


where, *μ* is the population mean; *a*_*i*_ is the additive effect of the i-th QTS with coefficient *x*_*ik*_, fixed effect; *aa*_*ij*_ is the additive-additive epistasis effect of the *i*-th QTS and the *j*-th QTS with coefficient *x*_*ik*_ · *x*_*jk*_, fixed effect; *e*_*h*_ is the main effect of the *h*-th environment, random effect; *ae*_*hi*_ is the additive by environment interaction effect of the *i*-th QTS and the *h*-th environment with coefficient *u*_*hik*_ (=*x*_*hik*_), random effect; *aae*_*hij*_ is the interaction effect of the *aa*_*ij*_ and the *h*-th environment with coefficient *u*_*hijk*_ (=*x*_*hik*_ · *x*_*hjk*_), random effect; and *ε*_*hk*_ is the random residual effect of the *k*-th line in the *h*-th environment.

In the present study, three association strategies including the GWA, QBA, and GBA, were adopted for data analysis. In the GWA and GBA, in order to alleviate heavy computation burden resulting from detection of the epistatic interactions for a large amount of SNPs (701,867 SNPs for GWA, and 294,498 filtered SNPs for GBA), the efficient parallel computation software GMDR-GPU^18^ was first employed for preliminary filtering of SNPs. In this process, both the marginal effects of each single SNP locus and interactions of each SNP pair were tested, which were achieved by separate genome scanning of one dimension (1D) and two dimension (2D), respectively. For each trait and each environment, ~1000 top candidate SNPs were screened by setting the “-m 1000” option in GMDR-GPU. Finally, 1212 and 841 SNPs were screened for the PH and HD, respectively, and used in the GWA; accordingly, 1063 and 914 SNPs for the PH and HD were screened and used in the GBA, respectively. In the case of QBA, 52,426 and 59,387 SNPs were directly used in association analysis for the PH and HD, respectively.

The software *QTXNetwork*^17^ was used for the association analysis. Firstly, a two-step mapping strategy was conducted during this procedure: 1) Individual locus detection. Based on the additive genetic model, significance testing was performed for each individual SNP locus by using *F*-test. The locus with *F*-statistic exceeding the threshold value was considered as a candidate individual SNP. 2) Epistasis loci detection. Using the significant SNPs detected in step 1 as covariates, a two dimension (2D) interaction scan was conducted for all possible SNP pairs by using *F*-test. The pair of loci with *F*-statistic larger than the threshold value is considered as candidate epistasis interacting loci. For the above two steps, the permutation testing was employed to determine the empirical threshold value of the *F*-statistic at the significance level of 0.05, being aimed to control the experiment-wise type I error rate. Then, model selection was performed using stepwise method based on *F*-test with each candidate locus and each candidate epistasis loci as selection unit; as a result, a final QTS full model like the Equation (1) could be established. Finally, all the parameters in model (1), as well as their standard errors and p-values, were estimated based on the samples generated by Markov chain Monte Carlo (MCMC) with 20,000 Gibbs sampler iterations, with the prior distributions that are same as described by Yang *et al*.^32^. The estimations and statistical inferences of the parameters were achieved by summarizing the Gibbs samples^17^.

## Additional Information

**How to cite this article**: Zhou, L. *et al*. Dissection of genetic architecture of rice plant height and heading date by multiple-strategy-based association studies. *Sci. Rep.* **6**, 29718; doi: 10.1038/srep29718 (2016).

## Supplementary Material

Supplementary Information

## Figures and Tables

**Table 1:** The estimated heritability and genetic effects of all significant SNPs for the plant height (PH) and the heading date (HD) through three analysis strategies.

Trait	QTS	Chr.	Allele	Strategy	Effect type	Effect size	−*log*_10_ (*P*)	*h*^2^ (%)	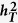 (%)
PH	rs25397447	1	A/G	GWA	a	2.61	10.74	8.91	45.63
	rs2745731	3	G/A	GWA	a	3.03	14.19	11.99	
	rs36929057	3	G/A	GWA	a	2.50	9.96	8.21	
	rs8819284	6	A/T	GWA	a	1.88	5.89	4.62	
	rs14788399	7	A/T	GWA	a	−1.83	5.60	4.37	
	rs4468159	11	C/T	GWA	a	−2.40	9.19	7.53	
	rs37194796	3	C/T	QBA	a	2.85	10.36	9.89	37.22
	rs9040677	6	T/A	QBA	a	2.34	7.23	6.69	
	rs9669459	6	T/C	QBA	a	2.24	6.65	6.10	
	rs14788399	7	A/T	QBA	a	−2.72	9.54	9.04	
	rs4468159	11	C/T	QBA	a	−2.12	6.06	5.50	
	rs24562025	1	G/A	GBA	a	2.20	6.55	5.72	40.19
	rs10179007	6	C/G	GBA	a	3.01	11.59	10.63	
	rs7478190	8	G/T	GBA	a	2.31	7.15	6.29	
	rs489068	12	C/T	GBA	a	2.63	9.04	8.14	
	rs10809004	12	C/A	GBA	a	−2.83	10.35	9.41	
HD	rs12807091	1	T/C	GWA	a	1.58	5.57	4.67	41.56
	rs22665688	1	C/T	GWA	a	2.29	10.89	9.71	
	rs26023433	1	C/T	GWA	a	1.41	4.54	3.71	
	rs3033880	6	A/T	GWA	a	−2.49	12.78	11.53	
	rs17909546	10	A/G	GWA	a	2.53	13.2	11.94	
	rs14583697	1	G/A	QBA	a	2.28	13.94	9.53	55.95
	rs4139855	4	T/C	QBA	a	2.22	13.26	9.03	
	rs2083119	5	C/T	QBA	a	−1.94	10.28	6.88	
	rs3070122	6	G/A	QBA	a	−2.51	16.73	11.55	
	rs14516781	7	A/T	QBA	a	2.02	11.09	7.46	
	rs13634598	10	G/A	QBA	a	1.78	8.75	5.78	
	rs17462383	10	T/C	QBA	a	1.77	8.66	5.72	
	rs22139712	1	G/A	GBA	a	1.91	7.64	7.08	37.53
	rs32347548	4	T/C	GBA	a	1.66	5.95	5.36	
	rs3112796	6	G/A	GBA	a	−1.78	6.77	6.19	
	rs12634148	10	C/T	GBA	a	1.45	4.67	4.09	
	rs17909546	10	A/G	GBA	a	2.24	10.31	9.79	
	rs457324	12	C/T	GBA	a	1.61	5.60	5.02	

QTS = the detected significant SNPs associated with the PH and the HD; Chr. = chromosome; Allele = paternal allele/maternal allele; GWA = genome-wide association, QBA = QTL-based association, GBA = gene-based association; a = additive effect for paternal allele homozygotes (QQ, ZH9308), -a for maternal allele homozygotes (qq, XQZB); −log_10_ (*P*) = inverse of the base 10 logarithm of p-values for significance of genetic effects; *h*^2^ (%) = heritability in percentage due to the genetic component effect; 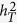 (%) = total heritability equal to summation of heritabilities of all individual QTSs.

**Table 2:** The estimated population means and total genetic values for ZH9308, XQZB, best line (BL) and superior pure line (SL) for the plant height and the heading date in three strategies.

Entry	Plant height (PH)			Heading date (HD)		
	GWA	QBA	GBA	GWA	QBA	GBA
Mean	81.55	81.40	80.61	98.49	98.33	99.46
ZH9308	5.79	2.58	7.33	5.32	5.62	7.08
XQZB	−5.79	−2.58	−7.33	−5.32	−5.62	−7.08
BL	14.25	12.27	12.98	10.31	14.52	10.64
SL	14.25	12.27	12.98	10.31	14.52	10.64

GWA = genome-wide association strategy; QBA = QTL-based association strategy; GBA = gene-based association strategy; Mean = estimated *μ* in Eq. (1); BL = best line that achieved the highest genetic value among the inbred lines; SL = the superior line with the potential highest genetic value in all designed homozygous lines.

**Table 3:** The annotation information of genes harboring the significant QTSs of plant height (PH) and heading date (HD).

QTS	Chr.	Allele	Trait	Gene ID	Gene Annotation
rs24562025	1	G/A	PH	LOC_Os01g43050	CENP-C1, putative, expressed
rs10179007	6	C/G	PH	LOC_Os06g17550	retrotransposon protein, putative, Ty3-gypsy subclass
rs7478190	8	G/T	PH	LOC_Os08g12640	retrotransposon protein, putative, Ty3-gypsy subclass, expressed
rs4468159	11	C/T	PH	LOC_Os11g08460	DnaK family protein, putative, expressed
rs10809004	12	C/A	PH	LOC_Os12g18700	expressed protein
rs489068	12	C/T	PH	LOC_Os12g01810	expressed protein
rs12807091	1	T/C	HD	LOC_Os01g22780	GDSL-like lipase/acylhydrolase, putative, expressed
rs22139712	1	G/A	HD	LOC_Os01g39330	helix-loop-helix DNA-binding domain containing protein, expressed
rs26023433	1	C/T	HD	LOC_Os01g45810	transposon protein, putative, unclassified, expressed
rs32347548	4	T/C	HD	LOC_Os04g54340	double-strand break repair protein MRE11, putative, expressed
rs4139855	4	T/C	HD	LOC_Os04g07784	transposon protein, putative, unclassified, expressed
rs3112796	6	G/A	HD	LOC_Os06g06610	expressed protein
rs12634148	10	C/T	HD	LOC_Os10g24620	retrotransposon protein, putative, Ty3-gypsy subclass, expressed
rs17909546	10	A/G	HD	LOC_Os10g33790	expressed protein
rs457324	12	C/T	HD	LOC_Os12g01750	zinc finger, C3HC4 type domain containing protein, expressed

Note: Gene annotation information comes from the database: MSU Rice Genome Annotation Project Release 7, http://rice.plantbiology.msu.edu/index.shtml.
