# Supplementary material for: Dissection of genetic architecture of rice plant height and heading date by multiple-strategy-based association studies
Source: Sci Rep. 2016 Jul 13;6:29718. doi: 10.1038/srep29718 (PMC4942822; doi:10.1038/srep29718)
Supplement: Supplementary Information [file srep29718-s1.docx]

**Dissection of genetic architecture of rice plant height and heading date by multiple-strategy-based association studies**

Liyuan Zhou, Shouye Liu, Weixun Wu, Daibo Chen, Xiaodeng Zhan, Aike Zhu, Yingxin Zhang, Shihua Cheng, Liyong Cao, Xiangyang Lou, and Haiming Xu

Supplementary Information

1. **Supplementary Figures**

Supplementary Figure S1**2**

Supplementary Figure S2**3**

Supplementary Figure S3**4**

1. **Supplementary Tables**

Supplementary Table S1**5**

Supplementary Table S2**6**

Supplementary Table S3**12**

Supplementary Table S4**13**

Supplementary Figure S1. LD heat maps for the 31 sigificant SNPs associated with either plant height or heading date.


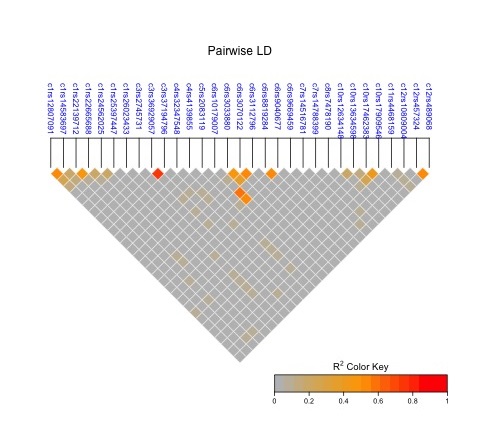


**Supplementary Figure S2**. Geographical map for two experimental locations with latitudes and longitudes.


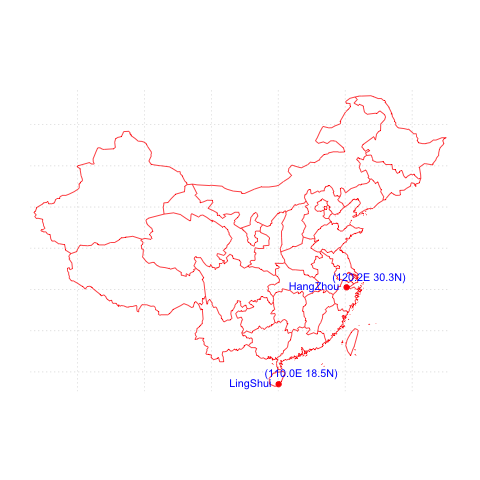


This figure was generated using R (v3.3.0) software with ‘maps (v2.2-6)’ and ‘mapdata (v3.1.0)’ packages. URL link, https://cran.r-project.org/.

Supplementary Figure S3. The histogram of phenotypic frequency distribution for plant height (PH) and heading date (HD) at each experimental location and all together.


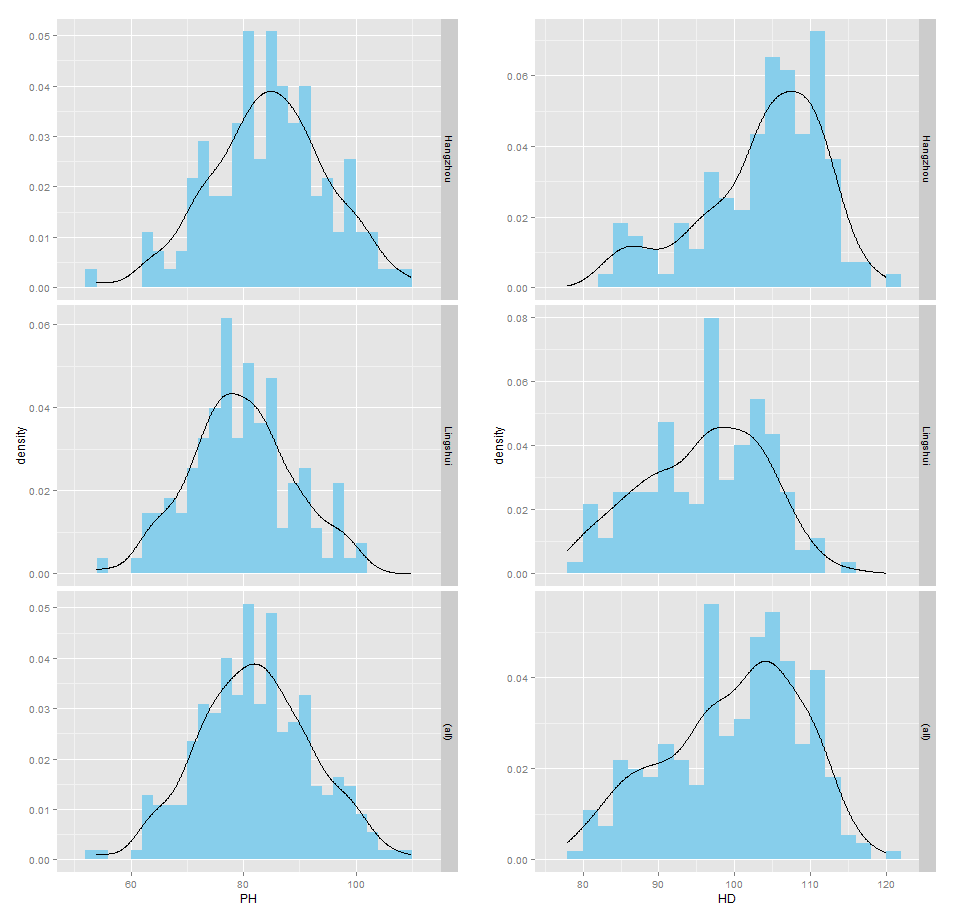


Supplementary Table S1. Summary statistics for plant height and heading date and their phenotypic correlation at each experimental location and all together.

| Entry | Plant height (PH) | | |  | Heading date (HD) | | |  | ρ(PH, HD) |
| --- | --- | --- | --- | --- | --- | --- | --- | --- | --- |
|  | Mean ± SD | Range | CV(%) |  | Mean ± SD | Range | CV(%) |  |  |
| Hangzhou | 84.0 ± 10.2 | 54.0 - 110.0 | 12.14 |  | 104.0 ± 8.1 | 83.0 - 120.0 | 7.79 |  | 0.63 |
| Lingshui | 80.0 ± 9.1 | 55.0 - 101.0 | 11.37 |  | 96.0 ± 8.0 | 78.0 - 115.0 | 8.33 |  | 0.47 |
| All | 82.0 ± 9.9 | 54.0 - 110.0 | 12.07 |  | 100.0 ± 8.9 | 78.0 - 120.0 | 8.90 |  | 0.59 |

Note: CV = coefficient of variation; ρ(PH, HD) = correlation coefficient between the PH and HD.

Supplementary Table S2. Details of sequencing and mapping data of each RI line and parents.

| **sample ID** | **Reads** | | | **Bases** | | | | | |
| --- | --- | --- | --- | --- | --- | --- | --- | --- | --- |
|  | **Total Reads** | **Mapped(%)** | **Unique Mapped(%)** | **Total Bases (Mb)** | **Mapping** | | **Unique Mapping** | | **MisMatch Bases** |
|  |  |  |  |  | **Mapped (%)** | **Mean Depth** | **Mapped (%)** | **Mean Depth** |  |
| ZH9308 | 78038190 | 88.59 | 69.26 | 6773 | 88.59 | 15.43 | 69.26 | 15.39 | 79 |
| XQZB | 66596780 | 87.73 | 70.12 | 5780 | 87.73 | 13.81 | 70.12 | 13.78 | 78 |
| s1 | 20879546 | 91.12 | 71.83 | 1693 | 91.12 | 5.27 | 71.83 | 4.64 | 15 |
| s2 | 11729318 | 90.44 | 70.13 | 951 | 90.44 | 3.3 | 70.13 | 2.88 | 8 |
| s4 | 26408204 | 91.17 | 72.61 | 2141 | 91.17 | 6.51 | 72.61 | 5.8 | 19 |
| s5 | 18478836 | 90.37 | 71.25 | 1498 | 90.37 | 4.71 | 71.25 | 4.16 | 13 |
| s10 | 14915848 | 91.66 | 73.15 | 1209 | 91.66 | 3.84 | 73.15 | 3.42 | 11 |
| s11 | 17176280 | 93.01 | 70.88 | 1376 | 93.01 | 4.23 | 70.88 | 3.6 | 15 |
| s12 | 9294444 | 90.62 | 71.18 | 753 | 90.62 | 2.67 | 71.18 | 2.37 | 7 |
| s13 | 25417292 | 92.44 | 69.91 | 2036 | 92.44 | 5.92 | 69.91 | 5.01 | 22 |
| s15 | 50310200 | 90.06 | 70.19 | 3484 | 90.06 | 8.08 | 70.19 | 7.16 | 25 |
| s21 | 15465826 | 92.47 | 69.56 | 1239 | 92.47 | 3.88 | 69.56 | 3.26 | 13 |
| s23 | 11440284 | 91.21 | 69.2 | 916 | 91.21 | 3.07 | 69.2 | 2.61 | 10 |
| s24 | 16544936 | 91.09 | 71.72 | 1341 | 91.09 | 4.28 | 71.72 | 3.77 | 11 |
| s25 | 13573672 | 90.02 | 71.88 | 1100 | 90.02 | 3.44 | 71.88 | 3.08 | 10 |
| s33 | 21469992 | 90.14 | 68.79 | 1720 | 90.14 | 4.96 | 68.79 | 4.26 | 18 |
| s35 | 15035538 | 90.63 | 71.2 | 1219 | 90.63 | 3.76 | 71.2 | 3.32 | 11 |
| s36 | 19101864 | 91.26 | 72.2 | 1548 | 91.26 | 4.6 | 72.2 | 4.07 | 13 |
| s39 | 24996982 | 90.66 | 71.96 | 2026 | 90.66 | 5.78 | 71.96 | 5.13 | 18 |
| s42 | 15851102 | 91.37 | 72.35 | 1285 | 91.37 | 3.94 | 72.35 | 3.49 | 11 |
| s43 | 13492564 | 91.04 | 72.25 | 1094 | 91.04 | 3.46 | 72.25 | 3.06 | 10 |
| s44 | 25797134 | 90.76 | 72.06 | 2091 | 90.76 | 5.92 | 72.06 | 5.27 | 19 |
| s47 | 19805426 | 91.08 | 71.78 | 1605 | 91.08 | 5.03 | 71.78 | 4.43 | 14 |
| s48 | 13088936 | 91.33 | 72.36 | 1061 | 91.33 | 3.48 | 72.36 | 3.09 | 9 |
| s49 | 16584136 | 91.11 | 71.53 | 1344 | 91.11 | 4.09 | 71.53 | 3.6 | 12 |
| s52 | 13351080 | 92.81 | 71.78 | 1070 | 92.81 | 3.42 | 71.78 | 2.96 | 12 |
| s59 | 19516368 | 90.38 | 71.37 | 1582 | 90.38 | 4.58 | 71.37 | 4.06 | 14 |
| s60 | 17125310 | 91.57 | 72.68 | 1388 | 91.57 | 4.32 | 72.68 | 3.82 | 12 |
| s61 | 18355548 | 91.6 | 69.17 | 1470 | 91.6 | 4.4 | 69.17 | 3.74 | 16 |
| s63 | 18267360 | 91.18 | 72.62 | 1481 | 91.18 | 4.43 | 72.62 | 3.95 | 13 |
| s67 | 18633002 | 90.5 | 71.16 | 1510 | 90.5 | 4.74 | 71.16 | 4.19 | 13 |
| s69 | 18114990 | 91.09 | 72.2 | 1468 | 91.09 | 4.66 | 72.2 | 4.12 | 13 |
| s71 | 13134930 | 90.74 | 72.19 | 1065 | 90.74 | 3.4 | 72.19 | 3.04 | 9 |
| s72 | 17436946 | 90.76 | 71.19 | 1413 | 90.76 | 4.5 | 71.19 | 3.95 | 12 |
| s73 | 14419070 | 91.72 | 69.45 | 1155 | 91.72 | 3.63 | 69.45 | 3.09 | 12 |
| s74 | 10362470 | 90.51 | 71.36 | 840 | 90.51 | 2.84 | 71.36 | 2.53 | 7 |
| s75 | 16905874 | 91.65 | 71.88 | 1370 | 91.65 | 4.44 | 71.88 | 3.89 | 12 |
| s77 | 15054500 | 90.89 | 71.77 | 1220 | 90.89 | 3.99 | 71.77 | 3.52 | 11 |
| s80 | 10762802 | 90.64 | 71.41 | 872 | 90.64 | 3.03 | 71.41 | 2.69 | 8 |
| s85 | 24683572 | 88.39 | 69.01 | 1989 | 88.41 | 5.5 | 69.03 | 4.78 | 18 |
| s86 | 15266440 | 91.47 | 71.75 | 1238 | 91.47 | 4.05 | 71.75 | 3.57 | 10 |
| s90 | 21787056 | 90.58 | 71.37 | 1766 | 90.58 | 5.13 | 71.37 | 4.54 | 16 |
| s93 | 14839412 | 90.78 | 71.64 | 1203 | 90.78 | 3.73 | 71.64 | 3.31 | 11 |
| s94 | 10876128 | 90.91 | 71.84 | 882 | 90.91 | 2.97 | 71.84 | 2.64 | 8 |
| s95 | 23326172 | 92.14 | 70.39 | 1869 | 92.14 | 5.47 | 70.39 | 4.69 | 20 |
| s98 | 12621256 | 91.63 | 69.59 | 1011 | 91.63 | 3.28 | 69.59 | 2.8 | 11 |
| s99 | 11577736 | 90.59 | 72.42 | 939 | 90.59 | 3.07 | 72.42 | 2.75 | 8 |
| s100 | 13242834 | 92.38 | 70.55 | 1061 | 92.38 | 3.38 | 70.55 | 2.88 | 11 |
| s101 | 16485576 | 90.68 | 71.78 | 1336 | 90.68 | 4.07 | 71.78 | 3.62 | 12 |
| s104 | 13926820 | 92.29 | 70.57 | 1116 | 92.29 | 3.55 | 70.57 | 3.04 | 12 |
| s108 | 8645922 | 90.81 | 72.16 | 701 | 90.81 | 2.63 | 72.16 | 2.35 | 6 |
| s109 | 18247260 | 91.34 | 72.64 | 1479 | 91.34 | 4.41 | 72.64 | 3.92 | 13 |
| s116 | 11754940 | 90.9 | 72.11 | 953 | 90.9 | 3.17 | 72.11 | 2.83 | 8 |
| s119 | 22590280 | 90.39 | 71.67 | 1831 | 90.39 | 5.31 | 71.67 | 4.72 | 16 |
| s120 | 26808168 | 90.55 | 70.17 | 2249 | 90.55 | 5.96 | 70.17 | 5.45 | 17 |
| s123 | 14552800 | 90.97 | 71.71 | 1180 | 90.97 | 3.75 | 71.71 | 3.32 | 10 |
| s125 | 11293658 | 92.16 | 73.59 | 915 | 92.16 | 3.23 | 73.59 | 2.87 | 8 |
| s128 | 13094700 | 92.16 | 66.79 | 1049 | 92.16 | 3.44 | 66.79 | 2.79 | 11 |
| s130 | 10308834 | 90.85 | 71.66 | 836 | 90.85 | 2.84 | 71.66 | 2.52 | 7 |
| s131 | 15112756 | 91.78 | 74.21 | 1225 | 91.78 | 3.79 | 74.21 | 3.42 | 11 |
| s132 | 11381704 | 91.18 | 72.42 | 923 | 91.18 | 3.21 | 72.42 | 2.85 | 8 |
| s133 | 23711856 | 91.23 | 71.64 | 1993 | 91.23 | 4.88 | 71.64 | 4.49 | 17 |
| s134 | 13340802 | 90.43 | 71.05 | 1081 | 90.43 | 3.61 | 71.05 | 3.19 | 10 |
| s139 | 10196982 | 91.51 | 72.15 | 827 | 91.51 | 3 | 72.15 | 2.66 | 7 |
| s141 | 21656676 | 92.68 | 70.57 | 1735 | 92.68 | 5.14 | 70.57 | 4.39 | 19 |
| s143 | 18174808 | 91.07 | 72.17 | 1473 | 91.07 | 4.48 | 72.17 | 3.97 | 13 |
| s147 | 17003020 | 91.09 | 71.85 | 1378 | 91.09 | 4.39 | 71.85 | 3.87 | 12 |
| s150 | 14310226 | 92.55 | 67.4 | 1146 | 92.55 | 3.69 | 67.4 | 3 | 12 |
| s151 | 11270824 | 91.22 | 72.44 | 914 | 91.22 | 3.07 | 72.44 | 2.73 | 8 |
| s152 | 19804176 | 90.66 | 70.91 | 1605 | 90.66 | 4.99 | 70.91 | 4.36 | 14 |
| s153 | 10149332 | 91 | 72.87 | 823 | 91 | 2.94 | 72.87 | 2.64 | 7 |
| s155 | 22444212 | 91.14 | 71.37 | 1819 | 91.14 | 5.4 | 71.37 | 4.72 | 16 |
| s158 | 11590780 | 92.37 | 70.53 | 929 | 92.37 | 3.12 | 70.53 | 2.67 | 10 |
| s160 | 7006164 | 91.42 | 72.44 | 568 | 91.42 | 91.42 | 72.44 | 3.97 | 5 |
| s164 | 18866574 | 91.79 | 73.31 | 1529 | 91.79 | 4.6 | 73.31 | 4.09 | 14 |
| s167 | 13249060 | 90.94 | 71.71 | 1074 | 90.94 | 3.48 | 71.71 | 3.06 | 9 |
| s168 | 24593828 | 91.8 | 70.94 | 2063 | 91.8 | 5.05 | 70.94 | 4.54 | 18 |
| s169 | 14679424 | 91.83 | 72.59 | 1190 | 91.83 | 3.77 | 72.59 | 3.32 | 10 |
| s173 | 14638556 | 89.92 | 70.89 | 1187 | 89.92 | 3.66 | 70.89 | 3.25 | 10 |
| s174 | 18524148 | 92.63 | 69.9 | 1484 | 92.63 | 4.43 | 69.9 | 3.72 | 16 |
| s175 | 19942968 | 92.2 | 69.81 | 1598 | 92.2 | 4.78 | 69.81 | 4.06 | 17 |
| s178 | 21842576 | 90.91 | 70.95 | 1771 | 90.91 | 5.5 | 70.95 | 4.82 | 15 |
| s181 | 25918122 | 90.31 | 70.8 | 2101 | 90.31 | 5.89 | 70.8 | 5.18 | 18 |
| s182 | 25117708 | 90.28 | 70.45 | 2103 | 90.28 | 5.97 | 70.45 | 5.65 | 9 |
| s184 | 29695200 | 91.56 | 69.27 | 2391 | 91.55 | 6.76 | 69.28 | 5.73 | 23 |
| s185 | 9927784 | 91.03 | 71.11 | 805 | 91.03 | 2.96 | 71.11 | 2.6 | 7 |
| s186 | 23239128 | 90.7 | 71.37 | 1884 | 90.7 | 5.56 | 71.37 | 4.92 | 17 |
| s190 | 9357580 | 90.37 | 71.63 | 759 | 90.37 | 2.76 | 71.63 | 2.46 | 7 |
| s192 | 13289058 | 91.21 | 70.44 | 1077 | 91.21 | 3.64 | 70.44 | 3.15 | 9 |
| s198 | 13084348 | 91.99 | 68.49 | 1048 | 91.99 | 3.43 | 68.49 | 2.87 | 11 |
| s199 | 11686906 | 89.83 | 71.19 | 947 | 89.83 | 3.08 | 71.19 | 2.75 | 8 |
| s201 | 28326984 | 91.63 | 72.64 | 2296 | 91.63 | 7.21 | 72.64 | 6.37 | 20 |
| s202 | 20299738 | 91.95 | 70.89 | 1626 | 91.95 | 4.81 | 70.89 | 4.13 | 18 |
| s203 | 20467606 | 90.43 | 68.22 | 1716 | 90.43 | 4.73 | 68.22 | 4.35 | 17 |
| s205 | 15904138 | 90.57 | 71.22 | 1289 | 90.57 | 3.96 | 71.22 | 3.51 | 12 |
| s211 | 20943550 | 92.22 | 74.15 | 1698 | 92.22 | 5.13 | 74.15 | 4.58 | 15 |
| s213 | 8778874 | 92.62 | 70.16 | 703 | 92.62 | 2.59 | 70.16 | 2.21 | 8 |
| s214 | 15637008 | 92.1 | 70.12 | 1253 | 92.1 | 3.88 | 70.12 | 3.31 | 13 |
| s217 | 12367786 | 92.91 | 69.84 | 991 | 92.91 | 3.3 | 69.84 | 2.77 | 11 |
| s219 | 13182804 | 90.95 | 71.21 | 1069 | 90.95 | 3.5 | 71.21 | 3.07 | 9 |
| s221 | 13420456 | 91.09 | 72.61 | 1088 | 91.09 | 3.54 | 72.61 | 3.16 | 9 |
| s222 | 16498512 | 92.34 | 67.81 | 1322 | 92.34 | 4.09 | 67.81 | 3.36 | 14 |
| s226 | 16208008 | 92.4 | 67.23 | 1298 | 92.4 | 4.07 | 67.23 | 3.31 | 13 |
| s227 | 38424540 | 91.44 | 71.18 | 3101 | 91.44 | 8.48 | 71.19 | 7.36 | 29 |
| s230 | 16593526 | 92.26 | 70.16 | 1329 | 92.26 | 4.05 | 70.16 | 3.45 | 14 |
| s231 | 15463624 | 90.54 | 70.92 | 1254 | 90.54 | 3.85 | 70.92 | 3.38 | 11 |
| s233 | 15453700 | 90.79 | 70.7 | 1253 | 90.79 | 3.89 | 70.7 | 3.39 | 11 |
| s237 | 18170388 | 89.82 | 71.5 | 1473 | 89.82 | 4.36 | 71.5 | 3.9 | 13 |
| s238 | 16796440 | 90.64 | 72.26 | 1362 | 90.64 | 4.09 | 72.26 | 3.66 | 12 |
| s240 | 14626992 | 92.75 | 70.75 | 1172 | 92.75 | 3.67 | 70.75 | 3.12 | 13 |
| s241 | 14789124 | 90.35 | 71.89 | 1199 | 90.35 | 3.7 | 71.89 | 3.28 | 10 |
| s242 | 14351306 | 91.71 | 69.67 | 1150 | 91.71 | 3.61 | 69.67 | 3.08 | 12 |
| s243 | 15375028 | 91.13 | 72.5 | 1246 | 91.13 | 3.84 | 72.5 | 3.41 | 11 |
| s245 | 20744972 | 91.14 | 72.37 | 1682 | 91.14 | 4.9 | 72.37 | 4.35 | 15 |
| s246 | 10463240 | 90.73 | 72.1 | 848 | 90.73 | 2.88 | 72.1 | 2.57 | 7 |
| s247 | 24660472 | 90.17 | 71.05 | 1999 | 90.17 | 5.65 | 71.05 | 5 | 17 |
| s248 | 15064004 | 90.3 | 71.34 | 1221 | 90.3 | 3.77 | 71.34 | 3.35 | 11 |
| s250 | 36970372 | 91.76 | 70.53 | 2974 | 91.75 | 8.17 | 70.53 | 7 | 30 |
| s252 | 17981222 | 91.46 | 72.51 | 1458 | 91.46 | 4.37 | 72.51 | 3.87 | 13 |
| s254 | 10596696 | 91.68 | 73.19 | 859 | 91.68 | 2.89 | 73.19 | 2.58 | 7 |
| s255 | 22108042 | 90.51 | 71.96 | 1792 | 90.51 | 5.23 | 71.96 | 4.66 | 16 |
| s256 | 17273094 | 90.8 | 71.61 | 1400 | 90.8 | 4.26 | 71.61 | 3.77 | 12 |
| s259 | 21468782 | 90.37 | 70.57 | 1740 | 90.37 | 5.19 | 70.57 | 4.56 | 15 |
| s260 | 15013718 | 91.15 | 71.68 | 1217 | 91.15 | 3.8 | 71.68 | 3.34 | 11 |
| s261 | 22182238 | 91.15 | 72.34 | 1798 | 91.15 | 5.22 | 72.34 | 4.64 | 15 |
| s263 | 15355858 | 90.29 | 71.83 | 1245 | 90.29 | 3.82 | 71.83 | 3.41 | 11 |
| s274 | 16261636 | 90.92 | 72.5 | 1318 | 90.92 | 3.96 | 72.5 | 3.54 | 12 |
| s278 | 16035336 | 92.56 | 70.24 | 1285 | 92.56 | 3.92 | 70.24 | 3.33 | 14 |
| s279 | 15010352 | 92.04 | 67.94 | 1202 | 92.04 | 3.83 | 67.94 | 3.17 | 12 |
| s280 | 22821544 | 91.41 | 71.81 | 1917 | 91.41 | 4.81 | 71.81 | 4.01 | 17 |
| s282 | 14722088 | 89.8 | 69.85 | 1193 | 89.8 | 3.69 | 69.85 | 3.2 | 10 |
| s284 | 11405798 | 92.61 | 70.47 | 914 | 92.61 | 3.05 | 70.47 | 2.6 | 10 |
| s285 | 18108700 | 92.76 | 70.68 | 1451 | 92.76 | 4.39 | 70.68 | 3.73 | 16 |
| s287 | 19910266 | 90.91 | 71.04 | 1614 | 90.91 | 5.08 | 71.04 | 4.44 | 14 |
| s289 | 19080106 | 91.96 | 67.45 | 1528 | 91.96 | 4.63 | 67.45 | 3.79 | 16 |
| s290 | 24457948 | 89.39 | 61.46 | 2052 | 89.39 | 5.04 | 61.46 | 4.53 | 18 |
| s295 | 17473752 | 90.81 | 72.7 | 1416 | 90.81 | 4.25 | 72.7 | 3.79 | 12 |
| s296 | 23016824 | 91.41 | 72.7 | 1866 | 91.41 | 5.7 | 72.7 | 5.05 | 16 |
| s298 | 11721440 | 90.95 | 71.74 | 950 | 90.95 | 3.1 | 71.74 | 2.75 | 8 |
| s299 | 22648518 | 92.27 | 70.7 | 1814 | 92.27 | 5.28 | 70.7 | 4.52 | 19 |

Supplementary Table S3. SNP markers distribution along each chromosome.

| **Chr.** | **Markers Distribution** | | | | | **Genes** | |
| --- | --- | --- | --- | --- | --- | --- | --- |
|  | **Total** | **CDS** | **5'UTR** | **Gene^a^** | **3'UTR** | **Number** | **Covered** |
| 1 | 42035 | 8307 | 719 | 17993 | 1372 | 6529 | 2747 |
| 2 | 49986 | 9217 | 990 | 13363 | 1744 | 5377 | 2629 |
| 3 | 61471 | 11876 | 1198 | 24595 | 2415 | 5568 | 2891 |
| 4 | 90111 | 19679 | 1071 | 16617 | 1619 | 5317 | 2756 |
| 5 | 60967 | 13120 | 1271 | 21120 | 2255 | 4572 | 2382 |
| 6 | 74999 | 13917 | 1234 | 27749 | 2516 | 4709 | 2661 |
| 7 | 55585 | 11492 | 892 | 37465 | 1546 | 4450 | 2547 |
| 8 | 47968 | 9096 | 612 | 27289 | 1111 | 4188 | 1976 |
| 9 | 81958 | 16916 | 1213 | 30508 | 1841 | 3407 | 2155 |
| 10 | 33412 | 6615 | 400 | 23600 | 676 | 3510 | 1755 |
| 11 | 64478 | 11975 | 841 | 19032 | 1425 | 4160 | 2539 |
| 12 | 38897 | 7947 | 568 | 35167 | 959 | 4014 | 2053 |
| All | 701867 | 140157 | 11009 | 294498 | 19479 | 55801 | 29091 |

^a^ Including gene loci and their alternative splicing isoforms.

Supplementary Table S4. Target QTLs used for filtration of SNPs in QBA with the corresponding literatures.

| Trait | QTL | Chromosome | Interval | Physical Position | Reference |
| --- | --- | --- | --- | --- | --- |
| PH | qPH-2 | 2 | RM250-RM166 | 32798753-35239676 | Xihong, S [^1^](#_ENREF_1) |
|  | qPH-6-1 | 6 | RM435-RM587 | 537354-2291931 |  |
|  | qPH-6-2 | 6 | RM6302-RM3330 | 8984566-10908028 |  |
|  | qPH-7 | 7 | RM180-RM2 | 5768455-16682897 |  |
|  | qPH-10 | 10 | RM271-RM6704 | 13161715-17490665 |  |
|  | qPH-10 | 10 | RM6142-RM271 | 12356665-13162025 |  |
|  | qPH-11 | 11 | RM167-RM4469 | 4057566-6182156 |  |
|  | qPH-2 | 2 | RM1920–RM263 | 25485848-25889855 | Liang, Y.S. et al.[^2^](#_ENREF_2) |
|  | qPH-7b | 7 | RM180–RM5436 | 5768455-9107949 |  |
|  | qPH-11 | 11 | RM1812–RM167 | 2392086-4057603 |  |
|  | qPH-3/qPH-3a | 3 | RM148-RM85 | 35835805-37198991 | Both[^1^](#_ENREF_1)^,^[^2^](#_ENREF_2) |
| HD | qHD-1-1 | 1 | RM1-RM3746 | 4635793-6155081 | Xihong, S[^1^](#_ENREF_1) |
|  | qHD-1-2 | 1 | RM23-RM5385 | 10753511-17306885 |  |
|  | qHD-4 | 4 | RM6770-RM8212 | 2813160-4415660 |  |
|  | qHD-5 | 5 | RM159-RM592 | 488027-2736697 |  |
|  | qHD-6-1 | 6 | RM19417-RM1163 | 2978412-4201396 |  |
|  | qHD-6-2 | 6 | RM136-RM6302 | 8763322-8984589 |  |
|  | qHD-6-2 | 6 | RM6302-RM3330 | 8984566-10908028 |  |
|  | qHD-7 | 7 | RM180-RM2 | 5768455-16682897 |  |
|  | qHD-8 | 8 | RM515-RM342 | 20048086-20279115 |  |
|  | qHD-10 | 10 | RM271-RM6704 | 13161715-17490665 |  |
|  | qHD-10 | 10 | RM6142-RM271 | 12356665-13162025 |  |
|  | qHD-2 | 2 | RM550-RM5812 | 12463506-15999886 | Liang, Y.S. et al.[^2^](#_ENREF_2) |
|  | qHD6b | 6 | RM6302-RM7213 | 8984566-10448545 |  |
|  | qHD7a | 7 | RM3670-RM2 | 13386382-16682897 |  |
|  | qHD7b | 7 | RM5436-RM3670 | 9107916-13386409 |  |
|  | qHD8 | 8 | RM342-RM32 | 20048086-20205005 |  |
|  | qHD-6-1/qHD6a | 6 | RM510-RM19417 | 2831543-2978433 | Both[^1^](#_ENREF_1)^,^[^2^](#_ENREF_2) |

Reference

1. Xihong, S. RIL Construction and QTL Mapping for Some Traits of Super Hybrid Rice (Oryza sativa L.),XY9308. (Chinese Academy of Agricultural Sciences, Hang Zhou, 2008).

2. Liang, Y.S. *et al.* Mapping of QTLs associated with important agronomic traits using three populations derived from a super hybrid rice Xieyou9308. *Euphytica* **184**, 1-13 (2012).
